# Precancerous cervical lesion in Ethiopia: systematic review and meta-analysis

**DOI:** 10.1186/s13643-021-01840-0

**Published:** 2021-11-01

**Authors:** Ayele Semachew Kasa, Tadesse Dagget, Yeshiwork Beyene, Getnet Dessie, Aklilu Endalamaw, Yinager Workineh, Emiru Ayalew, Balew Zeleke, Sitotaw Kerie, Getasew Mulat, Worku Animaw

**Affiliations:** 1 Department of Adult Health Nursing, College of Medicine and Health Sciences, Bahir Dar University, Bahir Dar, Ethiopia; 2 Department of Pediatrics and Child Health Nursing, College of Medicine and Health Science, Bahir Dar University, Bahir Dar, Ethiopia; 3 ALKAN Health Science Business and Technology College, Bahir Dar, Ethiopia

**Keywords:** Cervical cancer, Cervical lesion, Precancerous, Systematic review, Ethiopia

## Abstract

**Background:**

Though cervical cancer is largely preventable, it is still the second most common female cancer globally and the leading cause of cancer deaths among females in African. Though many efforts have been done to study the burden of the disease in Ethiopia, primary studies examining the prevalence of precancerous cervical lesions are fragmented. Hence, this systematic review and meta-analysis is aimed at estimating the pooled prevalence of precancerous cervical lesion and its trends in Ethiopia.

**Methods:**

This systematic review and meta-analysis was conducted using the following electronic databases. PubMed, Web of Science, SCOPUS, Science Direct, Google Scholar, African Index Medicus (AIM), African Journals Online databases, and Addis Ababa and Bahir Dar Universities research repositories were searched following the Preferred Items for Systematic Review and Meta-analysis (PRISMA) Guideline. STATA 15 statistical software was used to analyze the data. The quality of the included studies was assessed using the Joanna Briggs Institute (JBI) quality appraisal tool for meta-analysis. Heterogeneity between studies was assessed using the Cochrane *Q* test and *I*^2^ test statistics based on the random effects model. A random effects model was computed to estimate the pooled prevalence of precancerous cervical lesion in Ethiopia. Finally, the trend of precancerous cervical lesion in the country was presented.

**Result:**

Seventeen studies with a total of 26,112 participants were included in the analysis. The pooled prevalence of precancerous cervical lesion was 15.16 (*95% CI* 10.16–19.70). The subgroup analysis by region showed the highest prevalence of precancerous cervical lesion at the Southern Nations and Nationalities Peoples Region (19.65%; *95% CI* 15.51–23.80). The trend of precancerous cervical lesion prevalence showed an increased pattern over time.

**Conclusion:**

Approximately one among six of the study participants had precancerous cervical lesion. The trend also showed that there is still an increasing precancerous cervical lesion in Ethiopia. Best practices in achieving high vaccination coverage shall be informed by practices in other successful countries.

## Background

Cancers that originate in the female reproductive system are called women’s reproductive cancers. These include cancer of the cervix, breast, ovaries, vagina, vulva, and endometrium [1–3]. Though cervical cancer (CC) is largely preventable, it is still the second most common female cancer internationally and the leading cause of cancer deaths among females in African countries [4, 5]. Low levels of awareness and lack of effective screening programs, overshadowed by other communicable health priorities, are the possible factors for the observed higher incidence rate of cervical cancer in the developing regions of the world [1, 6].

Every year, approximately 500,000 new CC cases are diagnosed and 270,000 women die of this disease, mostly in developing countries [7, 8]. Globally, 86% of all CC diagnosed and 88% of CC deaths occur in developing regions of the world [9]. Cervical cancer is the second most frequently diagnosed cancer and the leading cause of cancer death in African women [10, 11].

The incidence is growing, as an estimated 570,000 new cases of CC was recorded in 2018, with approximately 90% of the deaths occurring in low- and middle-income countries [12]. Rates vary substantially across regions, with the incidence and death rates in East Africa and West Africa similar to rates in North Africa [13].

Reports of trends in CC mortality from less developed countries have been limited by poor data quality and inaccurate population estimates [14]. But because of poor access to quality screening and treatment service, the trend is increasing in these countries. According to trend analysis on CC between the years 1980 and 2010, the CC incidence rate was increased from 378,000 to 454,000 [15]. By 2020, it has been estimated that CC will be diagnosed in over 665,035 women worldwide, and 357,852 will die as a result [8].

In Africa, which has a population of 267.9 million women aged 15 years and older at risk of developing cervical cancer, approximately 80,000 women are diagnosed with cervical cancer per year, and just more than 60,000 women die from the disease [16]. Of the African burden of CC, the incidence and mortality in Sub-Saharan Africa are among the highest in the world and account for over 70% of the global CC burden with 70,000 new cases annually [8, 17].

According to the 2009 World Health Organization (WHO) report, the age-adjusted incidence rate of cervical cancer in Ethiopia was 35.9 per 100,000 patients with 7619 annual number of new cases and 6081 deaths every year [3, 18–20]. Other studies also showed that, of the nearly 22 million Ethiopian women over the age of 15, approximately 7600 are diagnosed with cervical cancer and roughly 6000 women die of the disease each year [18, 21–23].

Though many efforts have been done to study the burden of precancerous cervical lesion, primary studies reporting prevalence studies are fragmented. Previous studies in the literature have shown variations and discrepancies across different studies in Ethiopia. Hence, this systematic review and meta-analysis aims to consolidate data regarding the prevalence of precancerous cervical lesion in Ethiopia.

## Methods

### Reporting

The Preferred Reporting Items of Systematic Reviews and Meta-Analysis (PRISMA) checklist guideline was used to report the result of this systematic review and meta-analyses. In addition, the PRISMA flow chart was utilized to show the selection process of studies for a systematic review and meta-analysis [24].

### Searching strategies

This systematic review and meta-analysis was conducted to estimate the pooled prevalence of precancerous cervical lesion in Ethiopia. To conduct this study, all potentially relevant articles and gray literatures were meticulously searched. PubMed, Web of Science, SCOPUS, Science Direct, Google Scholar, African Index Medicus (AIM) and African Journals Online databases, and Addis Ababa and Bahir Dar Universities research repositories were searched using the following search terms: “Epidemiology,” “Burden,” “Magnitude,” “Prevalence,” “Cervical cancer,” “Cervix neoplasm,” “Cervix lesion,” “Pap Smear Positive,” “VIA Positive,” “Cervix precancerous lesion,” and “Ethiopia.” Search strings were developed using “AND” and “OR” Boolean operators. In addition to this, gray literature studies were searched from a research repository online library and a secondary search technique known as “footnote chasing” was utilized to identify additional articles from the included articles.

The search was carried out between August 28 and October 10, 2019, and all articles published until October 10, 2019, were included in the review.

### Inclusion and exclusion criteria

This systematic review and meta-analysis encompassed studies conducted only in Ethiopia using the English language. Research articles published in scientific journals and gray literature that reported the prevalence of precancerous cervical lesion were included in the review. Excluded studies included any which focused on the assessment of knowledge, attitude, and practice towards CC without the outcome of interest of this study, program evaluation studies, studies with only abstracts, case studies, and qualitative studies.

### Data extraction

Three authors (ASK, TD, and YB) extracted all necessary data by a standardized data extraction format using Microsoft Excel. The extracted parameters were primary author, publication year, region where the study was conducted, method of assessment, study design, mean age of the study participants, response rate, sample size, and prevalence of precancerous cervical lesion. Then, three authors (GD, AE, and YW) checked the data extraction process. Finally, five authors (BZ, EA, SK, GM, and WA) participated in approving the extraction process.

### Quality of the included studies

To appraise the quality of the included articles, we used the Joanna Briggs Institute (JBI) quality appraisal tool adapted for studies reporting prevalence data [25]. The following items were used to appraise cross-sectional studies: (1) inclusion criteria, (2) description of study subject and setting, (3) valid and reliable measurement of exposure, (4) objective and standard criteria used, (5) identification of confounder, (6) strategies to handle confounder, (7) outcome measurement, and (8) appropriate statistical analysis. Studies were considered low risk whenever fitted to 50% and/or above in the quality assessment checklist criteria [25, 26]. Using the aforementioned quality appraisal tools, no study was excluded (Table 1).

**Table 1:** Critical appraisal result of the included studies, 2019

Included articles	Criterion no. (items included to appraise cross-sectional and case-control studies)								
	1	2	3	4	5	6	7	8	%
Awoke et al. [27]	✓	✓	✓	✓	✓	✓	✓	✓	100
Belayneh et al. [28]	✓	✓	✓	X	✓	✓	X	✓	75
Birra [29]	✓	✓	✓	X	✓	✓	✓	✓	87.5
Gedefaw et al. [30]	✓	✓	✓	✓	✓	✓	✓	✓	100
Getinet et al. [31]	✓	✓	✓	✓	✓	✓	✓	✓	100
Hailemariam et al [32]	✓	✓	✓	✓	✓	X	✓	✓	87.5
Kassa et al. [33]	✓	✓	✓	✓	✓	✓	✓	✓	100
Kebede et al. [34]	✓	✓	✓	✓	✓	✓	✓	✓	100
Meseret and Tadiwos [35]	✓	✓	✓	✓	✓	✓	✓	✓	100
Netsanet et al. [36]	✓	✓	✓	✓	✓	✓	✓	✓	100
Pelzer et al. [37]	✓	X	✓	✓	X	X	✓	✓	62.5
Ruland et al. [38]	✓	✓	✓	✓	X	X	✓	✓	75
Sami-Ramzi et al. [39]	✓	✓	✓	✓	✓	✓	✓	✓	100
Teame et al. [40]	✓	✓	✓	✓	✓	✓	X	✓	90
Teka et al. [41]	✓	✓	✓	✓	✓	✓	✓	✓	100
Temesgen et al. [42]	✓	✓	✓	✓	✓	✓	✓	✓	100
Zewdie et al. [43]	✓	✓	✓	✓	✓	✓	✓	✓	100

√ criterion fulfilled, X criterion not fulfilled

Criterion no. 1: inclusion criteria, criterion no. 2: description of study subject and setting, criterion no. 3: valid and reliable measurement of exposure, criterion no. 4: objective and standard criteria used, criterion no. 5: identification of confounder, criterion no. 6: strategies to handle confounder, criterion no. 7: outcome measurement, and criterion no. 8: appropriate statistical analysis

### Data analysis

The data were analyzed using STATA version 15 Statistical Software. Heterogeneity across studies was checked using the inverse variance (*I*^*2*^) and Cochran *Q* statistics. The cut-offs of 25%, 50%, and 75% were used to declare the heterogeneity as low, moderate, and severe, respectively [44, 45].

As the preliminary output of the test statistics revealed a significant heterogeneity among studies (*I*^2^ > 70%, *P*< 0.05), a random effects model was used to estimate the pooled prevalence of precancerous cervical lesion with a 95% confidence interval (*CI*). Subgroup analysis was also performed based on study area (regions) and human immunodeficiency virus (HIV) status in relation to the outcome variable. Funnel plot, Egger, and Begg tests at 5% significant level were employed to assess publication bias [46, 47].

## Results

### Description of the identified studies

**Fig. 1:**
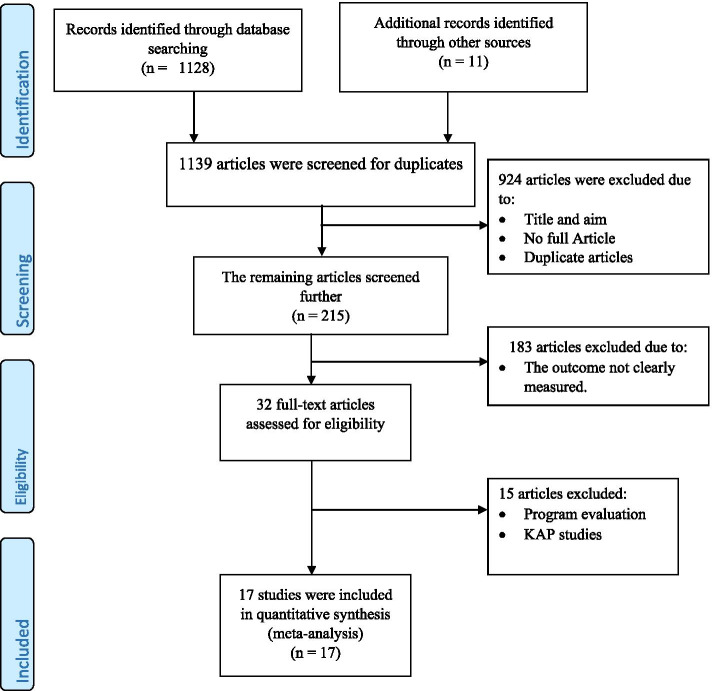
PRISMA flow diagram showing the selection of studies for a systematic review and meta-analysis, 2019

Up to October 10, 2019, we identified a total of 1139 articles using different databases, Addis Ababa and Bahir Dar University research repositories. Nine hundred twenty-four articles were excluded because of duplication and relevance-related issues. Among the remaining 215 articles, 183 articles were excluded because the outcome was not clearly measured. Fifteen articles were excluded because they were program evaluation and knowledge, attitude, and practice-related reports. Finally, 17 studies fulfilled the eligibility criteria and were included in the final systematic review and meta-analysis (Fig. 1).

### Overview of included studies

Among the total 17 studies included in this current review, five of them were from Addis Ababa City Administration [29, 35–37, 40]**.** Five studies were included from the Southern Nations Nationalities and People Region (SNNPR) [30, 32, 38, 39, 41]. In addition, five studies were included from the Amhara region [27, 28, 31, 33, 42]. The remaining two articles were from Oromia [43] and Tigray [34]. Most of the studies used VIA as the measure of assessment for the outcome variable. The total participants in the included studies were 26,112 with samples ranging from 189 [38] to 16,632 [36]. The response rate of all the included studies was greater than 90%, and the quality score of the studies ranged from 62.5 to 100% (Table 2).

**Table 2:** Characteristics of the included studies in the review and meta-analysis, Ethiopia, 2019

Author name	Study year	Region	Method of assessment	HIV status	Study design	Mean age	Response rate (%)	Sample size	Total *N* outcome	Prevalence (%)	Quality score
Awoke et al. [27]	2017	Amhara	VIA	Both	Cross-sectional	35	100%	428	61	14.3	100
Belayneh et al. [28]	2018	Amhara	Pap smear	HIV	Cross-sectional	34.48	97.60%	284	28	9.9	75
Birra [29]	2016	Addis Ababa	VIA	NS	Cross-sectional	35.73	99%	316	89	28.4	87.5
Gedefaw et al. [30]	2013	SNNPR	VIA	HIV	Cross-sectional	33	98%	448	99	22.1	100
Getinet et al. [31]	2014	Amhara	Pap stain	Both	Cross-sectional	35.02	97.80%	400	56	14.1	100
Hailemariam et al. [32]	2010	SNNPR	Diagnosis logbook	Both	Cross-sectional	41.6	91.70%	2120	350	16.5	87.5
Kassa et al. [33]	2015	Amhara	Women’s chart	HIV	Cross-sectional	35.9	100.00%	435	88	20.2	100
Kebede et al. [34]	2017	Tigray	VIA	Both	Cross-sectional	32.95	100%	342	23	6.7	100
Meseret and Tadiwos [35]	2015	Addis Ababa	VIA	NS	Cross-sectional	35.7	99%	226	54	24.1	100
Netsanet et al. [36]	2014	Addis Ababa	VIA	HIV	Cross-sectional	NR	100%	16632	1663	10	100
Pelzer et al. [37]	1992	Addis Ababa	Cytological investigation	NS	Cross-sectional	NR	90%	2111	33	1.56	62.5
Ruland et al. [38]	2006	SNNPR	Digene HPV test	NS	Cross-sectional	34	100%	189	30	15.9	75
Sami-Ramzi et al. [39]	2006	SNNPR	VIA	NS	Cross-sectional	NR	100.00%	537	93	17.3	100
Teame et al. [40]	2016	Addis Ababa	VIA	Both	Cross-sectional	39.87	95%	360	46	12.8	90
Teka et al. [41]	2017	SNNPR	Women’s chart	Both	Cross-sectional	NR	100%	528	146	27.7	100
Temesgen et al. [42]	2019	Amhara	VIA	Both	Cross-sectional	36.26	100%	422	29	6.9	100
Zewdie et al. [43]	2013	Oromia	VIA	Both	Cross-sectional	32.4	100.00%	334	43	12.9	100

*NS* not stated, *VIA* visual inspection through acetic acid

### Publication bias

**Fig. 2:**
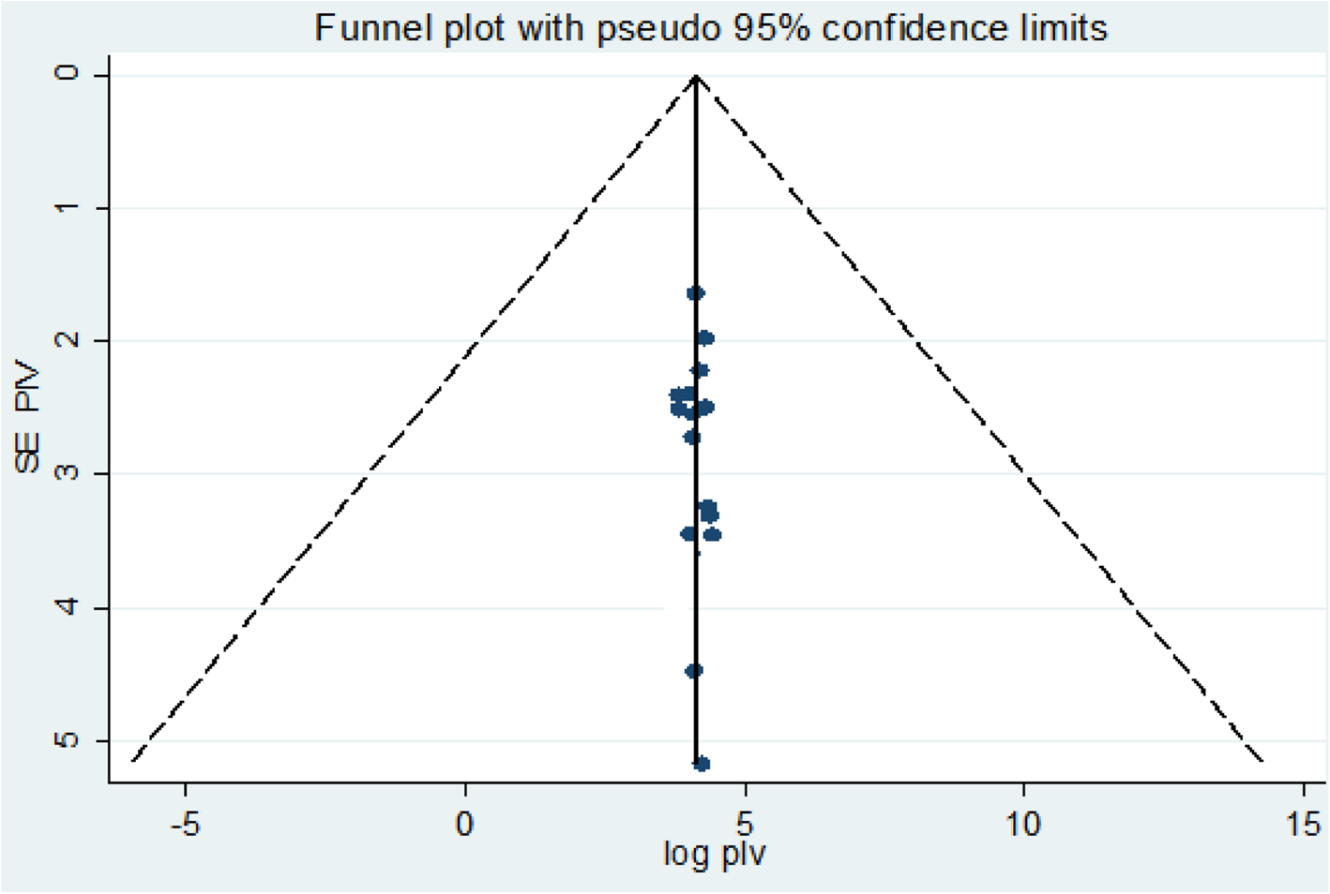
Funnel plot presentation to assess publication bias for precancerous cervical lesion in Ethiopia: systematic review and meta-analysis, 2019

To assess publication bias, both the funnel plot and the Egger’s test were conducted in the meta-analysis. The visual examination of the funnel plot exhibited a symmetric distribution of studies (Fig. 2). In addition to the funnel plot, Egger’s regression test was (*β* = −0.0063, *SE*=0.08, *P*=0.88) showing no statistical evidence of publication bias for the included studies.

### Prevalence of precancerous cervical lesion

**Fig. 3:**
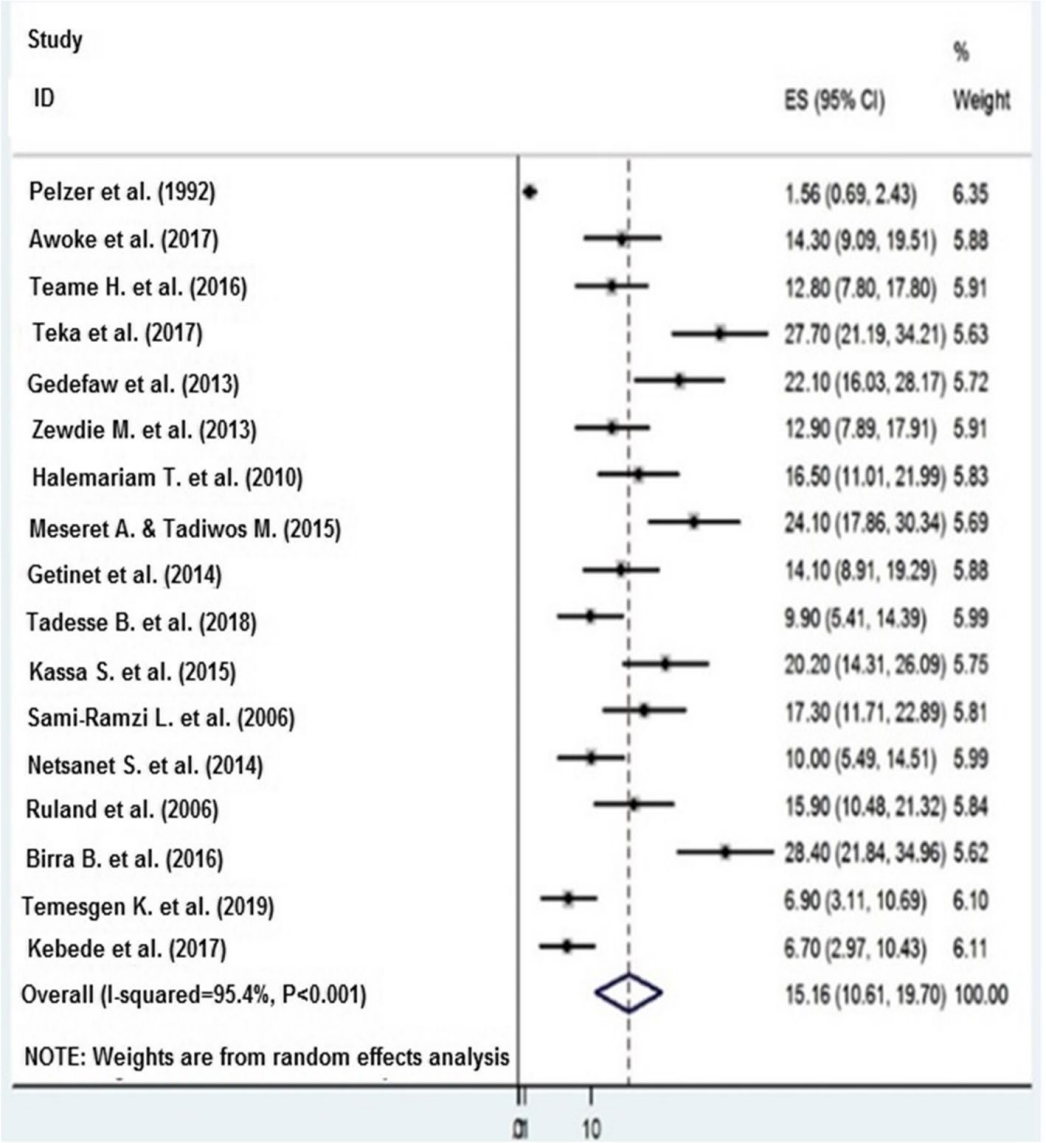
Pooled prevalence of precancerous cervical lesion in Ethiopia, 2019

The lowest prevalence of precancerous cervical lesion was 1.56% which is reported from SNNPR [37] whereas the highest prevalence was 28.4% [29] reported from a study done in Addis Ababa. Overall, the pooled prevalence of precancerous cervical lesion in Ethiopia was 15.16 (*95 CI* 10.16–19.70) (Fig. 3).

*I* ^2^ statistic test for heterogeneity indicated that the studies differed significantly (*I*^2^= 95.40%, *p* < 0.001). Hence, we used a DerSimonian and Laird random effects model [48, 49] to estimate the pooled prevalence of precancerous cervical lesion. Studies that showed the largest weight were Plezer et al. [37] that showed 6.35, Kebede et al. [34] with 6.11, Temesgen et al. [42] with 6.10, and Belayneh et al. [28] that showed 6.80 weight, whereas Birara et al. showed a relatively smaller weight which was 5.62 (Fig. 3).

Despite having one of the lowest single study prevalence rates, the highest pooled subgroup analysis of precancerous cervical lesion prevalence was found in SNNPR, 19.65% (*95% CI* 15.51–23.80), followed by Addis Ababa City Administration which the pooled prevalence was 15.10 (*95% CI* 4.77–25.44).

An additional subgroup analysis was done using the HIV status of study participants. Accordingly, the highest pooled estimate of precancerous cervical lesion was found in those study participants whose sero-status is unknown, 17.27% (*95% CI* 5.12–29.41). However, the pooled prevalence rate of precancerous cervical lesion in those study participants whose status was confirmed to be HIV-positive was 15.27% (*95% CI* 9.06–21.48) (Table 3).

**Table 3:** Subgroup analysis showing the prevalence of precancerous cervical lesion among Ethiopian regions, 2019

Variables	Region/city administration	Number of studies	Sample size	Estimate (*95% CI*)
By region	Addis Ababa	5	19,645	15.10 (4.77, 25.44)
	SNNPR	5	3822	19.65 (15.51, 23.80)
	Amhara	5	1969	12.76 (8.36, 17.16)
	Other*	2	676	9.56 (3.51, 15.62)
By HIV status	Unknown/undiagnosed	5	3379	17.27 (5.12, 29.41)
	Both (HIV+ and HIV−)	8	4934	13.63 (9.47, 17.79)
	HIV+	4	17,799	15.27 (9.06, 21.48)
Overall		17	26,112	15.16 (10.61, 21.48)

The meta-regression analysis was conducted considering publication years, HIV status, sample size, and study area. However, the results showed that none of these variables was a statistically significant source of heterogeneity.

### Trend analysis

**Fig. 4:**
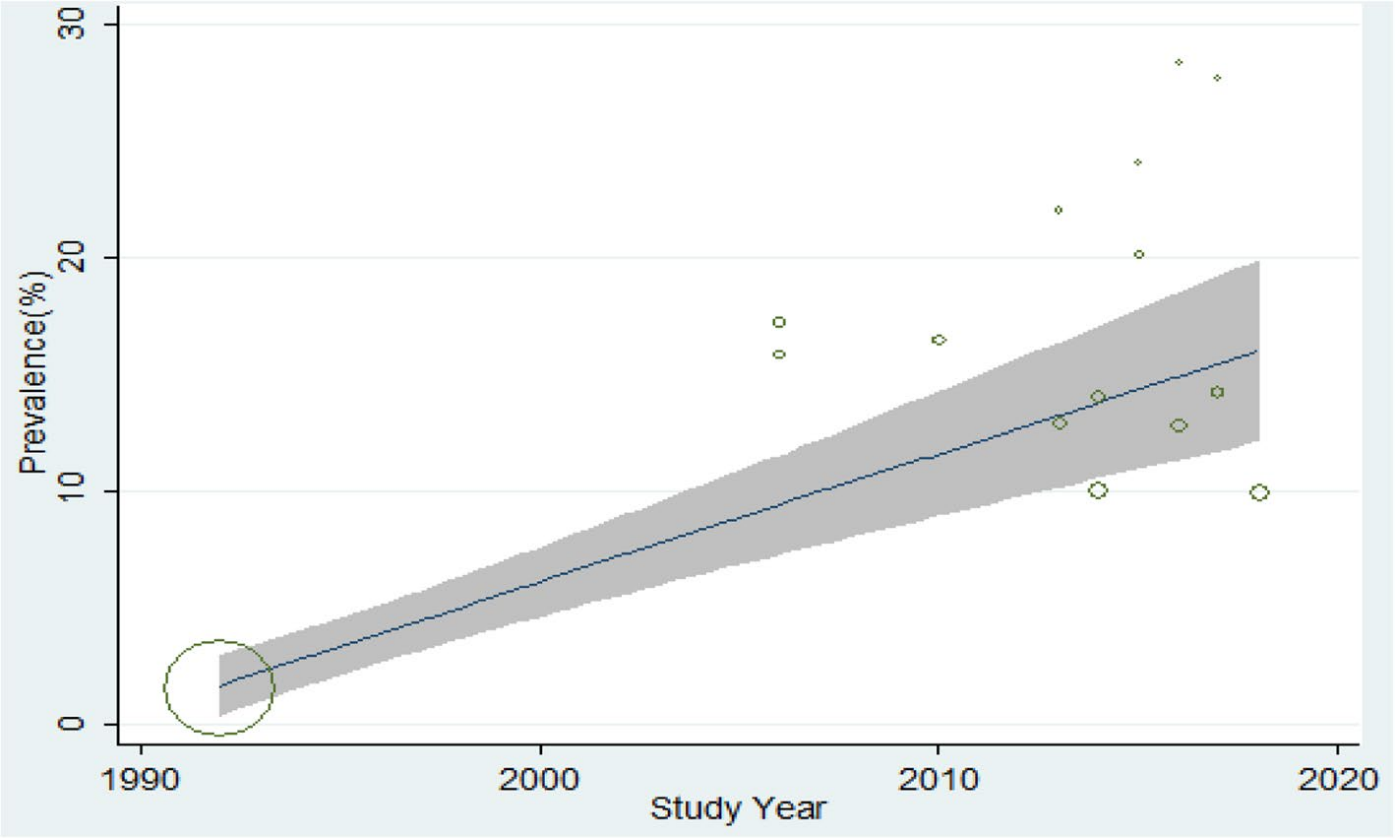
Trends of precancerous cervical lesion in Ethiopia using prevalence and year of study (*B* = 0.56, *P* = 0.014)

Trends of the prevalence of precancerous cervical lesion were done to observe the patterns of the disease in Ethiopia using the reported prevalence and year of study. A significant upward trend in the prevalence of precancerous cervical lesion was observed from 1992 to 2019 (*B* =0.56, *P*=0.014) (Fig. 4).

## Discussion

Promoting CC awareness and the importance of screening has been advocated in Ethiopia since 2008 [50]. Despite this fact, very few women receive screening services [51]. The current systematic review and meta-analysis aimed to estimate the pooled prevalence of precancerous cervical lesion among Ethiopian women between 1992 and 2019, providing information about trends and demographics. The result revealed that the pooled prevalence of precancerous cervical lesion was 15.16% (*95% CI* 10.16, 19.70). This finding is almost consistent with a study done in Rural Nigerian women of which 16.6% had precancerous cervical lesion [52]. A facility-based cross-sectional study of mostly HIV+ women from Tanzania and another study from Nigeria revealed 17% and 17.8% women, respectively, had precancerous cervical lesion [53, 54].

Our meta-analysis shows higher prevalence than from a systematic review done in Tanzania that showed the overall prevalence of precancerous cervical lesion was 9.2% [55] and a study from Zaria State in Nigeria in which the overall prevalence of precancerous cervical lesion was 4.8% [56]. Another study done in Kwara State, Nigeria, showed only 5% of study participants had precancerous cervical lesion [57]. A community-based screening in Turkey showed a precancerous cervical lesion prevalence of 9.4% precancerous cervical lesion [58]. A similar study in India using a community-based cervical cancer screening program among women of Delhi obtained 4.67% precancerous cervical lesion prevalence [59].

The possible explanation for this discrepancy might be the mean age of marriage among the respondents in the aforementioned studies was relatively higher. As early marriage is one of the risk factors for having precancerous cervical lesion, this might augment the incidence and prevalence of the precancerous cervical lesion. The other explanation might be most of researches conducted in the aforementioned countries were from urban settings and they might have awareness and access to information and this might lead them to have early screening for the disease.

This finding is lower than a study done in Senegal that revealed 21.03% of study participants had precancerous cervical lesion [60]. Another study done in Nigeria among HIV-positive women showed that 22.2% of women had precancerous cervical lesion [61]. The possible elucidation for such discrepancy between the current finding and other comparable study findings might be due to the difference in the socio-demographic variations in the included study participants. A study from countries contained a data collected mostly from the rural population and HIV-positive women while in our study, both urban and rural settings were considered. The other possible explanation for the above variation could be due to the difference in study design.

Based on the subgroup analysis, the highest pooled prevalence of precancerous cervical lesion was seen in Southern Nation and Nationalities and People’s Region (SNNPR) (19.65%). This finding was higher compared to studies conducted in Addis Ababa and Amhara region that revealed 15.10% and 14.35%, respectively. The possible explanations for this variation might be accounted by variations in information dissemination across the regions for reproductive health women about the disease. Hence, those women who had no information might remain with the symptom/disease. Understanding the reasons for these variations will include comparing regional methods of information dissemination related to reproductive health and disease. All of these can also be compared to interventions in countries with lower prevalence rates, in order to identify best practices, and promising interventions appropriate for implantation in Ethiopia.

It is known that HIV infection is one of the main risk factors for the development of cervical dysplasia. On this regard, there are many reports on the association of HIV with increased risk of cervical dysplasia [54, 61–63]. However, the current systematic review and meta-analysis in the subgroup analysis revealed that the highest precancerous cervical lesion (17.27%) was found in those study participants whose sero-status was unknown. This finding is supported by the WHO report that stated the likely pattern of precancerous cervical lesion expected in a previously unscreened population of women [31, 33, 64, 65]. In addition to this, the possible explanation for this finding might be accounted by participants who were not screened for HIV might be exposed for other risk factors for cervical cancer. Moreover, though their sero-status was not known at the time of precancerous cervical lesion screening, they might previously know that their sero-status for HIV/AIDS was positive.

### Limitation

In the current systematic review and meta-analysis, we noticed the following limitations. The pooled prevalence which was determined using the eligible 17 studies only represented four different administrative regions in Ethiopia. Hence, it does not represent the whole country as studies in more than half of the regions were not eligible for inclusion in the current review. Utilization of various data collection tool in the included studies might have its own influence in generalizing the finding. In addition, potentially unpublished studies were not considered and latent sources of heterogeneity may also have an impact in the overall finding of the result.

## Conclusion

The current study revealed that one in six study participants were positive for precancerous cervical lesion. The study also showed that there is an increased trend of precancerous cervical lesion prevalence in the country. Possible ways to increase awareness programs may include using various social media. In addition, best practices in achieving high vaccination coverage should be considered from other successful countries.

## Data Availability

All data are available in the manuscript.

## Abbreviations

CC: Cervical cancer
*CI*: Confidence interval
HIV: Human immuno virus
HPV: Human papilloma virus
JBI: Joanna Briggs Institute
VIA: Visual inspection with acetic acid
VILI: Visual inspection with Lugol’s iodine
SNNPR: Southern Nations Nationalities and People Region
WHO: World Health Organization
